# Strong grain neighbour effects in polycrystals

**DOI:** 10.1038/s41467-017-02213-9

**Published:** 2018-01-12

**Authors:** Hamidreza Abdolvand, Jonathan Wright, Angus J. Wilkinson

**Affiliations:** 1 Department of Mechanical and Materials Engineering, Western University, Spencer Engineering Building, London, ON N6A 5B9 Canada; 2 Department of Materials, University of Oxford, Parks Road, Oxford, OX1 3PH UK; 3 ESRF, 71 Avenue Des Martyrs, 38000 Grenoble, France

## Abstract

Anisotropy in single-crystal properties of polycrystals controls both the overall response of the aggregates and patterning of local stress/strain distributions, the extremes of which govern failure processes. Improving the understanding of grain–grain interactions has important consequences for in-service performance limits. Three-dimensional synchrotron X-ray diffraction was used to study the evolution of grain-resolved stresses over many contiguous grains in Zr and Ti polycrystals deformed in situ. In a significant fraction of grains, the stress along the loading axis was found to decrease during tensile plastic flow just beyond the macroscopic yield point; this is in the absence of deformation twinning and is a surprising behaviour. It is shown that this phenomenon is controlled by the crystallographic orientation of the grain and its immediate neighbours, particularly those adjacent along the loading axis.

## Abstract (web summary)

Understanding how individual crystals share load inside a polycrystal is crucial to improve component lifetime, but remains difficult to measure. Here, the authors show that the crystal orientation of a grain and that of its neighbours can surprisingly cause stress relaxation in zirconium and titanium under load.

## Introduction

Due to their unique mechanical properties, hexagonal closed-packed (HCP) polycrystals such as zirconium, titanium and magnesium are extensively used in nuclear, aerospace, and transportation industries. Understanding the mechanism of load sharing between grains of HCP polycrystals is a current major research area that aims to tailor materials microstructure for improved life spans^1–3^. For instance, the interaction and load sharing between the grains of titanium alloys can lead to the phenomenon known as cold dwell fatigue that limits the life span of commercial aerospace components^4^. Likewise, such interactions in zirconium alloys control the process of delayed hydride cracking in the key core components of nuclear reactors^5^. The prediction of component safe service conditions and lifetimes requires accurate materials models that can capture evolution of stress fields at multiple length scales.

The engineering performance of these polycrystals is significantly affected by the elastic and plastic anisotropy of the constituent single crystals. Such anisotropy can potentially result in highly localised stress fields that can evolve into crack nucleation sites. For instance, the accommodation of plastic deformation by the movement of dislocations on basal or prism slip systems results in formation of the slip bands that induce large localized stress fields close to grain boundaries^6^. Since the driving force for activating such dislocation movement is relatively low, grains with high basal or prism slip activity are known as 'soft' grains^7^. For these grains, the misorientation between basal plane normal and the loading direction (*β*) is generally high. Plastic deformation along the 〈*c*〉 axis of the HCP crystal, on the other hand, requires much higher driving force and is often accommodated by twinning which induces a large lattice rotation^8,9^. Such grains are known as 'hard' grains and possess a low *β* angle.

While recent experimental measurements and modeling studies have revealed large localized stress fields near the tips of twins^10–12^, it is generally believed that twin transformation strain is one of the key factors that can relax grains resolved stresses in HCP alloys^13^. For instance, with the use of neutron diffraction it was shown that formation of twins results in stress relaxation in the texture component selected by the diffraction of an incident beam^14,15^. These studies suffered from the uncertainty in measuring the initial crystal parameters of newly generated twins and the consequent uncertainty in measuring the relaxation. With the use of three-dimensional synchrotron X-ray diffraction (3D-XRD), the single-crystal parameter problem was removed and by measuring all of the stress tensor components, it was shown that twins are indeed relaxed^16–19^. These stress relaxations have also been reported for shape memory alloys undergoing martensitic phase transformation. Similarly, it is believed that the transformation strain associated with phase transformation in such alloys controls the plateau observed in their stress–strain curves^20–22^. More recently, with the use of 3D-XRD, it was shown that the modified localized stress field at the front of transformation zone is responsible for such relaxation and controls the nucleation of the new martensitic phase^23^.

3D-XRD is a powerful diffraction technique that uses hard X-ray to determine the centre-of-mass (COM) position, average elastic strain, stress, lattice orientation and relative volume of each grain in the probed volume^23,24^. The calculation of grains COMs provides a unique opportunity to study the effects of grain neighbourhood on the load sharing between hard and soft grains.

In this paper, 3D-XRD and crystal plasticity finite element (CPFE) modelling techniques are used to study fundamentals of deformation in commercially pure zirconium (CPZr) and titanium (CPTi). We show that while macroscopic stress increases, even in the absence of twinning, stress relaxation at the grain level still exists and is solely a result of the crystal anisotropy and load sharing between grains of the polycrystals. This is significant for accurate model development and extracting the driving force, i.e., critical resolved shear stress (CRSS), for activating particular slip or twinning systems. This is evidence of grain level stress reduction during dislocation-mediated plastic flow in HCP polycrystals, a phenomenon that, to the best of our knowledge, was previously undocumented.

## Results

### The 3D-XRD experiments and CPFE models

**Fig. 1:**
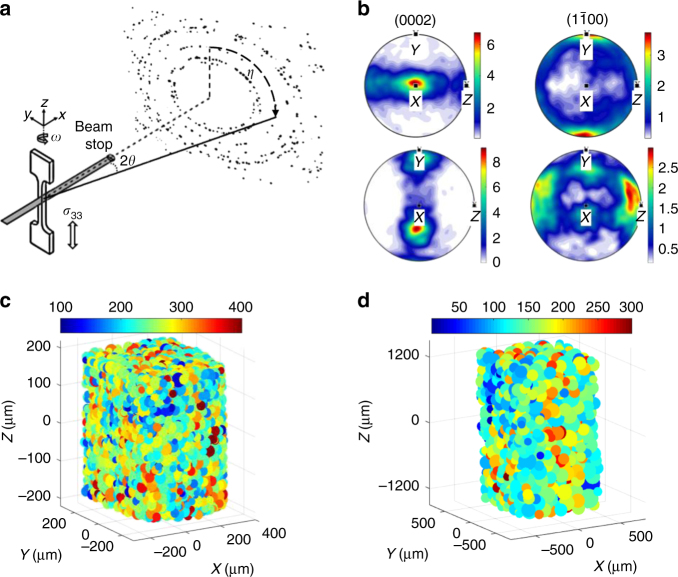
The setup and the results of the 3D-XRD experiments on CPZr and CPTi. **a** A schematic of the 3D-XRD experimental setup. Results of 3D-XRD: **b** measured pole figures (in mrd) for CPZr (top) and CPTi samples (bottom). Distribution of *σ*_33_ (in MPa) at the maximum applied strain for (**c**) CPZr and (**d**) CPTi

The chemical composition of the CPZr and CPTi samples are provided in Supplementary Table 1. These samples were firstly annealed at 700 °C and then air cooled to relieve as far as possible residual stresses, though anisotropy in the thermal expansion coefficients means these cannot be eliminated. Both samples were then mechanically polished and chemically electro-polished until high-quality Kikuchi patterns could be obtained using an electron backscatter diffraction (EBSD) setup. An example of the EBSD map measured for the undeformed sample is shown in Supplementary Fig. 1. Once the samples were polished, the cross sections were measured to be 0.5 × 0.53 mm^2^ for CPZr and 0.91 × 0.97 mm^2^ for CPTi. The 3D-XRD experimental setup and the coordinate system used in this paper are shown in Fig. 1a, where the *z*-axis coincides with the loading direction, the *x*-axis points into the sample thickness along the incident X-ray beam direction. The 3D-XRD measured pole figures of the samples are shown in Fig. 1b, which show that most of the grains have their *c*-axis oriented towards the *x*-axis with smaller population oriented towards the loading direction for the CPZr sample.

The CPZr sample was mechanically loaded in situ while diffraction measurements were carried out at the preload, onset of plasticity (~0.6% strain), maximum applied strain of 1.2% and finally unload. The CPTi sample was loaded in the same way but with the maximum applied strain of 0.94%. Both samples were deformed under strain control at an applied strain rate of 2.64 × 10^−5^ s^−1^. Details of the 3D-XRD experimental setup and the steps taken for post-processing diffraction patterns are provided in the Methods section. At each measurement step, samples were firstly strained and then re-aligned to ensure the same volume was probed. As shown in Table 1, macroscopic stress relaxation occurs while the sample is held at a fixed total strain mostly during the re-alignment step but to a much lesser extent also during the diffraction measurement. On average, the re-alignment step took 40 min. Measurement of diffraction patterns for CPTi and CPZr samples happened over 3 and 7.5 h, respectively, during which time negligible macroscopic stress relaxation was observed. CPZr had longer measurement time at each loading step as >11,000 grains were measured in a 0.375 mm length of the probed gauge, whereas just 2100 CPTi grains were measured over 2.4 mm of the gauge. The statistics of 3D-XRD experiments for each sample are provided in Table 1. The 3D-XRD measured relative volumes were used as the weight function for calculating grain-weighted average stresses in these two tables. Also, maximum average errors for grain centre of masses belong to the plastic zone and are 6.7 and 6.8 μm for CPZr and CPTi samples, respectively. An example of the measured grain-resolved stresses in the loading direction for CPZr and CPTi samples are shown in Fig. 1c, d.

**Table 1:** Details of the 3D-XRD experiment of the CPZr and CPTi samples

CPZr												
		Measured macro stress (MPa)			Number of grains	Average number of peaks per grain	Grain-weighted average stress with estimated errors (MPa)					
Step	Applied strain (%)	Before alignment	After alignment	End of measurement			*σ*_*xx*_	*σ*_*yy*_	*σ*_*zz*_	*σ*_*xy*_	*σ*_*xz*_	*σ*_*yz*_
Preload	0.0	7.5	7.5	7	11,247	102	−20 ± 27	8 ± 19	2 ± 16	−1 ± 4	−2 ± 5	2 ± 7
Onset of plasticity	0.59	350	287	258	10,677	102	−23 ± 27	11 ± 20	249 ± 17	−2 ± 4	2 ± 5	1 ± 7
Plastic zone	1.2	374	304	270	8869	99	−24 ± 33	13 ± 24	264 ± 20	−2 ± 5	2 ± 7	2 ± 8
Unload	NA	11	11	10	9013	98	−28 ± 33	9 ± 24	2 ± 21	0 ± 5	−2 ± 7	1 ± 8
**CPTi**												
Preload	0.0	11.3	11.3	11.3	2184	93	3 ± 30	4 ± 23	15 ± 19	−1 ± 6	1 ± 6	−4 ± 10
Onset of plasticity	0.39	165	135	124	1749	85	5 ± 26	10 ± 20	126 ± 32	−2 ± 4	0 ± 5	−5 ± 7
Plastic zone	0.94	198	152	143	1694	83	13 ± 41	16 ± 30	148 ± 26	−3 ± 7	0 ± 8	−5 ± 13
Unload	NA	2.7	2.7	2.7	1728	85	12 ± 39	15 ± 28	5 ± 24	0 ± 7	0 ± 8	−6 ± 12

CPFE modelling was used to simulate deformation of CPZr and CPTi. The details of the mathematical model are given in the Methods section. Generally, at the beginning of each time step, total strain, rotation and time increments are provided by the ABAQUS finite element (FE) solver to our user subroutine^25^. Depending on the current state of the RSS on each slip system, shear increment can be calculated from which the plastic strain and rotation increments can subsequently be calculated. The elastic strain increment, and hence stress increment, can then be calculated by deducting the plastic strain increment from the total strain increment. Since rotation increments are calculated in the subroutine, it is possible to investigate the evolution of grain orientations. We use this property to confirm whether the 〈a〉 pyramidal slip system is active in CPZr.

The measured COMs and relative volumes of grains were used to simulate grain shapes and import the simulated microstructure into the FE solver. This was done by first calculating the physical volume of each grain in the scanned volume assuming that grains are space-filling. The weighted Voronoi technique developed in Abdolvand et al.^19,26^ was used to mesh the grains. In this technique, a cube of elements is firstly generated to determine the position of each element in the cube. Equation (1) is then used for grain assignment to each element:

1 $$C_i = \left\{ {{\bf{X}} \in {\bf{R}}^{\bf{d}}\left| {\left\| {{\bf{X}} - {\bf{s}}_{\bf{i}}} \right\|^2 - w_i^2 < } \right.\left\| {{\bf{X}} - {\bf{s}}_{\bf{j}}} \right\|^2 - w_j^2,i \ne j} \right\},$$

where **X** is the position vector of the element in the cube, **s**_**i**_ is the position vector of the seed point of the grain *i*, and *w*_*i*_ is the radius of the same grain. The physical meaning of Eq. (1) is that for a given element *E* located at the position **X**, *E* should be assigned to the grain *i*, which has closest seed point **s**_**i**_ to *E* comparing to any other grains in the cube.

**Fig. 2:**
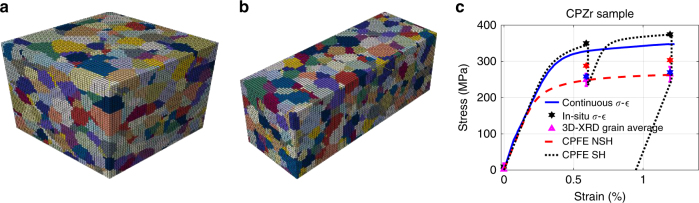
Simulated microstructures and comparison between stress–strain curves. Simulated microstructures of (**a**) CPZr, and (**b**) CPTi samples. These microstructures were used for CPFE modelling. **c** Comparison between measured and simulated macroscopic stress–strain curve for CPZr; black, red and blue stars represent the maximum load, beginning and end of diffraction measurement for each step

Since it is computationally costly to simulate heterogeneous deformation of all of the measured grains, only a subset of grains was imported into the FE solver. For the CPZr sample, 1038 of the grains that fall into a cube of 200 µm side length were simulated. This cube was meshed at a step size of 4 µm. For the CPTi, 994 grains that fall into a cuboid of 800 × 800 × 2200 µm^3^ were simulated, with meshing implemented at a step size of 25 µm. The simulated microstructures for CPZr and CPTi samples are shown in Fig. 2a, b, respectively. At room temperature, plastic deformation of CPZr and CPTi is mostly controlled by prism $\left\langle {11\bar 20} \right\rangle$, basal $\left\langle {11\bar 20} \right\rangle$ and pyramidal $\left\langle {11\bar 23} \right\rangle$ slip systems. Tensile or compression twins can also form if the load is applied along the *c*-axis of the HCP crystals^25^. In this study, the majority of the grains have their *c*-axis perpendicular to the loading direction and EBSD measurement of the deformed samples confirmed the absence of twinning (Supplementary Fig. 2), so the effect of twinning is neglected.

The results of the FE convergence tests are provided in Supplementary Figs. 3 and 4. In Fig. 2c, the results of FE simulation for average stress–strain curve of CPZr are compared with the measured values. The continuous *σ*−*ε* curve was measured ex situ at slightly slower strain rate (10^−5^ 1 s^−1^). The in situ measured values for each step are also shown. The three stars represent the measured stresses by the load cell at the maximum load, after alignment, and after diffraction measurements. The 3D-XRD grain-weighted stresses for the probed volume are also shown. Grain-measured relative volumes were used as the weight factors. Two models are used to generate the results; for the first model, the effects of strain-hold (SH) during experiment was considered. For the second model, no strain-hold (NSH) was applied, i.e., CPTi and CPZr samples were continually deformed to the maximum applied strain.

For model SH, the single-crystal parameters extracted by Abdolvand et al.^25^ and provided in Supplementary Table 2 were used; the effects of plastic hardening are considered for the SH model, whereas for model NSH, the CRSS values reported by Gong et al.^27^ were simply reduced to account for the relaxation during strain holds. These values for prism, basal and pyramidal slip systems for the CPZr sample in model NSH are 82, 109 and 287 MPa, respectively, with no further hardening^27^; for the CPTi sample they are 70, 140 and 210 MPa, respectively^28^. The rate sensitivity parameters for both NSH and SH are the same and provided in Supplementary Table 2.

### General trends

**Fig. 3:**
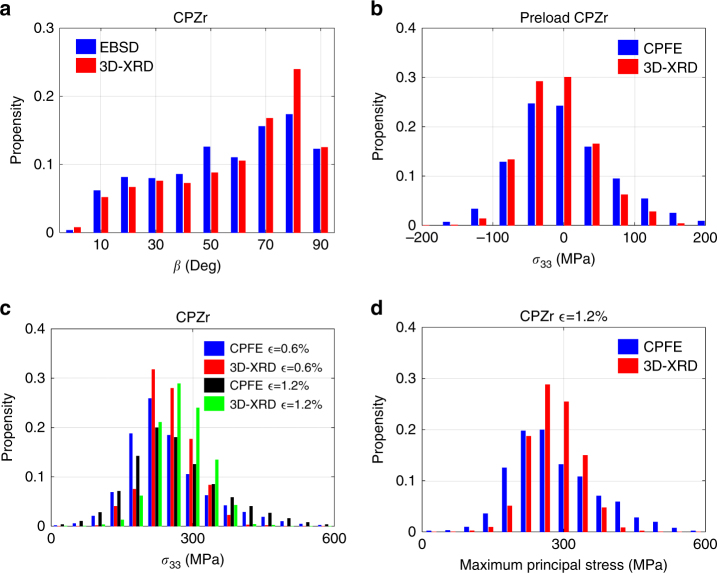
General trends. **a** The propensity of misorientation (*β*) between basal plane normal and the loading direction measured by 3D-XRD and EBSD. Comparison between 3D-XRD measured and CPFE-NSH simulated values for *σ*_33_ (**b**) at the preload and (**c**) applied strain of 0.6 and 1.2%. **d** Measured and calculated maximum principal stresses

Histograms of *β* angles between loading axis and crystal *c*-axis, measured by 3D-XRD and EBSD indicate that the CPZr texture gives a higher probability of the *c*-axis being perpendicular to, rather than parallel with the loading axis, as is shown in Fig. 3a. The very good agreement validates the two independent measurement methods. The distributions of 3D-XRD measured *σ*_33_ stress along the load axis at the preload, onset of plasticity and maximum applied strain are shown in Fig. 3b, c and are compared against results of CPFE model for the CPZr sample. It is shown in Fig. 3b that grains of CPZr sample are already stressed at the preload step. CPFE simulation confirms that this is due to the thermal strains that develop during annealing. The annealing was done at 700 °C followed by slow cooling to room temperature, but due to the anisotropy in coefficient of thermal expansion and crystal elastic modulus, residual stresses as high as 200 MPa can develop. In Fig. 3c, the comparison of *σ*_33_ at the onset of plasticity and applied strain of 1.2% shows that as the sample average *σ*_33_ increases, the distribution of *σ*_33_ broadens which is an indication of plastic deformation at the grain level. The full-width at half maximum (FWHM) of the measured *σ*_33_ at the onset of plasticity is 99.5 MPa while at the applied strain of 1.2% it is 122.5 MPa. Since at the grain level, stresses are tensorial, rather than uniaxial, the distribution of the maximum principal stress is shown in Fig. 3d. The rest of the stress components are shown in Supplementary Figs. 5 and 6. A good agreement between the FE model and 3D-XRD measurements was found.

### Analysis of slip system activity

**Fig. 4:**
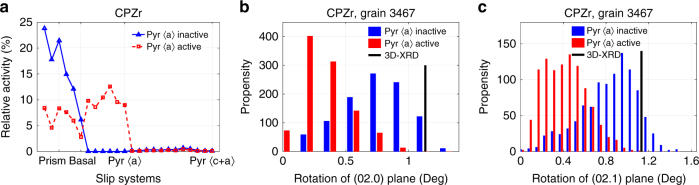
Effects of 〈a〉 pyramidal slip activity. **a** Relative activity of all grains for the two examined CPFE models. Comparison between measured and calculated rotations of (**b**) (02.0) and (**c**) (02.1) planes for the two examined models

There has been some discussion in the literature about whether pyramidal 〈a〉 slip makes any significant contribution to deformation of alpha Ti and Zr alloys at room temperature. The 3D-XRD data were used in combination with the CPFE simulations to assess the likely need to include this slip system in addition to prism 〈a〉, basal 〈a〉 and pyramidal 〈c + a〉. Simulations were made using the NSH model for CPZr with and without inclusion of pyramidal 〈a〉 slip, which was given a low CRSS value matched by that of prism 〈a〉 slip (82 MPa). The calculated cumulative shears on each slip system accommodated at 1.2% of applied strain for these models are shown in Fig. 4a, from which it is clear that given the available crystal orientations, significant activity would be expected where this a viable deformation mode. Relative activities are calculated based on how much of the calculated plastic shear strain is accommodated on each slip system relative to the total calculated plastic shear. Since in both models lattice rotation is allowed (Eqs. (5)–(7)), the calculated rotation matrices at the end of 1.2% strain were used to calculate the rotation of the most affected peaks. Figure 4b, c shows the distribution of angular tilting of the (02.0) and (02.1) plane normals calculated using lattice rotations from simulations with and without the inclusion of 〈a〉 pyramidal slip for the grain identified as having the greatest shear accumulated on the 〈a〉 pyramidal slip system. Our calculation shows that the {02.0} plane families are the most affected planes by such slip activity. Result shown in Fig. 4 indicates that the inclusion of 〈a〉 pyramidal slip system in the model would result in mis-calculation of crystal rotations. Hence, all of the results provided following this section are with 〈a〉 pyramidal slip system switched out.

### Grain stress relaxation

**Fig. 5:**
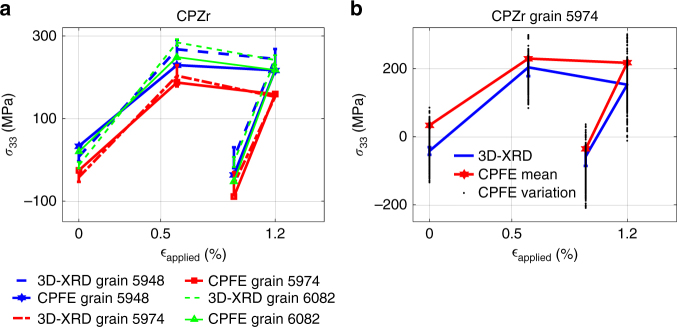
Examples of *σ*_33_ stress drop observed in some selected grains. **a** Average measured and calculated *σ*_33_, and (**b**) variation of *σ*_33_ calculated at each integration point assigned to grain 5974

Evidence for strong grain neighbour effects is observed for both CPTi and CPZr samples. In both cases while the macroscopic stress increases from the onset of plasticity to the maximum applied strain, in many of the grains, the stress along the loading direction (*σ*_33_) actually decreases. Examples of the phenomenon are shown in Fig. 5. Different levels of stress drop are observed for different grains. This is also captured in the CPFE modelling. Using the constructed Voronoi-based microstructure, it is possible to identify the adjoining neighbouring grains for every crystal in the scanned volume.

**Fig. 6:**
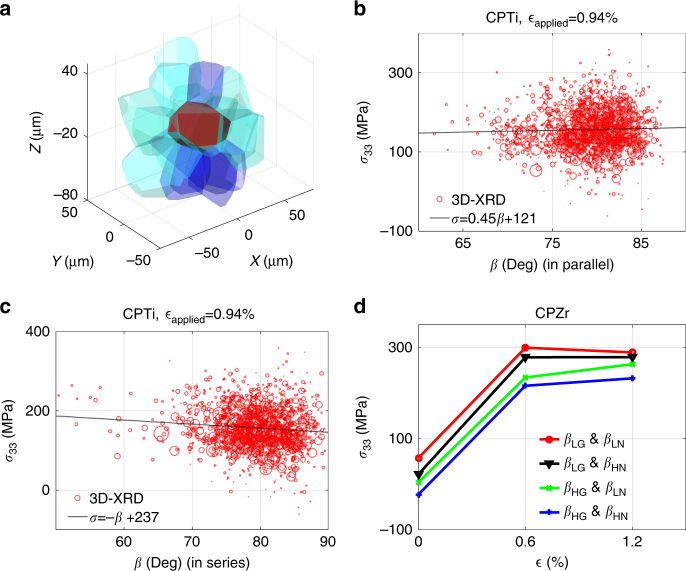
Effects of grain neighbourhood. **a** An example of parallel and serial grains: dark and light blue represent grains adjacent in *z*-direction and *x*–*y* plane, respectively. *σ*_33_ as a function of *β* averaged over neighbours for CPTi grains (**b**) in parallel and (**c**) in series. The size of each circle is proportional to the measured grain relative volume. **d** 3D-XRD results for load sharing between grains. *β* represents the angle between the loading direction and the crystal *c*-axis, subscripts HG, LG and 'N' represent low *β*, high *β* and neighbouring grain, respectively

The behaviour of a grain and its local neighbourhood tends to differ depending on whether they are loading and deforming in parallel or in series with the central grain. For *σ*_33_ this means differentiating between grain neighbours along the *z*-axis (in series), from those lying close to the *x*–*y* plane through the grain centroid (in parallel). This means neighbouring grains in series are closer to the central grain along the *z*-axis in comparison to *x*–*y* plane, whereas grains in parallel are closer to the central grain in the *x*–*y* plane. The illustrative example in Fig. 6a shows these two groups of neighbouring grains in different colours. Loading in parallel with hard stiff grains should lead to load shedding and stress reduction in the central grain, while soft neighbouring grains should increase the stress. In contrast, one expects hard grains in series to resist deformation leading to increased imposed strain, and thus often increases stress on the central grain. In the following, we partition the neighbouring grains into two groups one acting in series with the central grain, and the others that act in parallel.

The variation of *σ*_33_ measured with 3D-XRD as a function of the *β* angle at maximum load in CPTi and for the neighbouring grains acting in parallel and series are, respectively, shown in Fig. 6b, c. The *β* angle on the *x*-axis represent the weighted average of *β*s for the neighbours of the central grain. Complementary results for the neighbouring grains acting in parallel and series are shown in Supplementary Fig. 7. Although significant scatter is observed, there is a clear underlying trend. When neighbours act in parallel with the central grain, we expect a neighbourhood with high *β* to encourage greater tensile stress in the central grain. Such a trend can be seen in the 3D-XRD data shown in Fig. 6b, where a line with the slope of 0.45 MPa per ° indicates the level of correlation. On the other hand, grains in series should show the opposite trend and this is indeed captured by 3D-XRD measurements (Fig. 6c). The effect is stronger here with the correlation line through the data have a gradient of −1.0 MPa per °. To further evaluate the influence of grain neighbourhood on local load sharing, grains were divided into four categories: grains with low *β* and neighbours in series with low average *β* (*β*_LG_ and *β*_LN_), grains with low *β* but neighbours in series with high average *β* (*β*_LG_ and *β*_HN_), grains with high *β* but neighbours in series with low average *β* (*β*_HG_ and *β*_LN_), and grains with high *β* and neighbours in series with high average *β* (*β*_HG_ and *β*_HN_). In Fig. 6d, the evolution of stress for the four categories measured by 3D-XRD on CPZr is shown. It is shown that hard grain in series with hard neighbours (*β*_LG_ and *β*_LN_) has the most obvious stress drop among all while possess the highest *σ*_33_ stress. For this case, hard grains are surrounded by hard neighbours and the interaction resulted in a stress drop during plastic deformation despite the overall sample hardening.

**Fig. 7:**
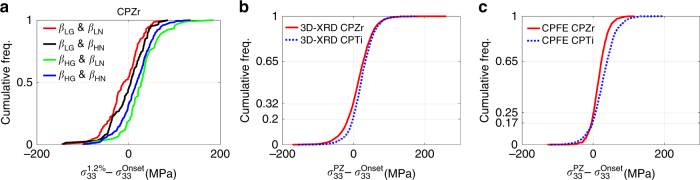
Stress drop in CPTi and CPZr samples. Cumulative frequency of stress drop: (**a**) for different grain neighbours, **b** for all of the measured grains with their corresponding CPFE results shown in **c**. *β* represent the angle between loading direction and the crystal *c*-axis, subscripts HG, LG and N represent low *β*, high *β* and neighbouring grain, respectively. PZ refers to plastic zone where maximum strain of 1.2 and 0.94% was applied to CPZr and CPTi samples, respectively

The empirical cumulative distribution function (cumulative freq.) of *σ*_33_ stress change for each grain neighbour category is shown in Fig. 7a. For a given stress variation of Δ*σ*, cumulative frequency represents the fraction of grains in the sampled volume that experience a stress drop equal or <Δ*σ*. It is shown that although a stress decrease is observed for some grains in each category, the most significant stress drops belongs to hard grain surrounded by hard neighbours (*β*_LG_ and *β*_LN_), where more than 50% of the grains experienced a stress drop. To further analyse the unusual stress drop, the collective behaviour of all of the measured grains of CPZr and CPTi samples are studied and are shown in Fig. 7b. More than 5000 CPZr and 1300 CPTi grains are used for calculating the cumulative frequencies. These selected grains could be tracked from preload to unload. It is clear that the *σ*_33_ stress drop is observed for both samples, where 32% and 20% of CPZr and CPTi grains, respectively, experienced it. CPFE results for the cumulative frequency of the stress drop for both samples are also shown in Fig. 7c. There is a striking agreement between CPFE and 3D-XRD results, where 25% and 17% of CPZr and CPTi grains, respectively, experienced a stress drop. This is purely a result of elastic and plastic anisotropy of the HCP crystal and strong grain–grain interactions. The anisotropic indices, ratio between Eigen values of the elastic tensor for CPZr are [0.78, 1.11, 1.47] whereas that of CPTi are [1.06, 0.75, 1.21]^13^. That is why fewer CPTi grains experience such a stress drop.

## Discussion

**Fig. 8:**
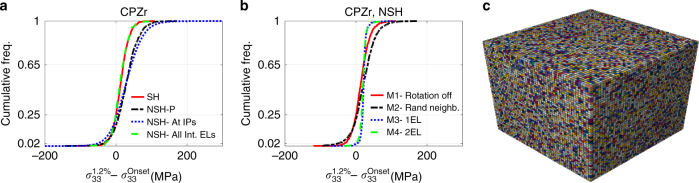
Modelling results for cumulative frequencies of stress drops. **a**, **b** Cumulative frequency of stress drop for different cases examined by CPFE modelling. SH represents strain-hold model and NSH represents the model with no strain hold. In the NSH-P model, parameters reported in Abdolvand et al.^25^ were used. **c** An example of self-consistent type model with fragmented grains, where random positions were assigned to fragments. Each colour represents an orientation

To better understand the source of the observed stress drop, various assumptions were tested using the CPFE modelling results of which are given in Fig. 8. All of the CPFE results shown so far were for NSH model for which CRSS values of the active slip systems were reduced to account for the stress relaxation that happened during each measurement step. To study the effects of single-crystal parameters, those parameters calibrated by Abdolvand et al.^25^ using more than 120 lattice strains measured through a series of neutron diffraction experiments on a dilute alloy of Zr were used in the CPFE-SH model. These parameters are given in Supplementary Table 2. For this model, the effect of displacement hold was considered. A very good agreement between measured and calculated macroscopic stress–strain curve was achieved, which is shown in Fig. 2c. Average stresses within individual grains at the end of each measurement were then calculated, from which stress drops were then calculated. The cumulative frequency of the stress drop for the SH model is shown in Fig. 8a. Similar to the NSH model, more than 25% of the grains experienced a stress drop, however for the SH model, it is not possible to discern if the source of the stress drop is the relaxation that happens during the displacement hold or the grain–grain interaction. Hence, the same single-crystal parameters were used in the model NSH to investigate the possible stress drop, with the results shown in Fig. 8a. This model is called NSH-P and it was observed that more than 17% of the grains experienced stress drop. This is lower than the NSH model with reduced CRSS values and no hardening, yet high enough to reinforce that the observed stress drop does not originate from the stress relaxation that occurs during the displacement hold. All of these observations point to strong grain neighbour effects.

On average, more than 950 integration points (IPs) were assigned to each grain of the CPZr model, which is significantly in excess of the 350 IPs shown to be a minimum that should be used to capture heterogeneous distribution of stress within a grain^29^. Although average stress within grains is studied, the possible contribution of IPs at the grain boundaries to the stress drop was also investigated to ensure that the observed stress drop in CPFE modelling was not simply a numerical artefact. In the first case, the stress drop at each IP was used to calculate the cumulative frequency; this is labelled NSH-AT IPs in Fig. 8a. More than 22% of IPs experienced a stress drop. In the second case, all of the elements located at the grain boundaries or at the surface of the model were removed from calculating average stress within grains to give the cumulative frequency distribution NSH-All Int Els in Fig. 8a. More than 25% of grains still experienced a stress drop, which is consistent with the rest of our CPFE results. In our CPFE modelling, 1036 CPZr grains were modelled where 266 of the modelled grains experience *σ*_33_ stress drop. Since in simulating grain morphologies, the volumes of the grain were considered (Eq. (1)), more elements were assigned to bigger grains. It is observed that more than 50% of the grains that experienced stress drop possess more than 350 IPs, which further confirms the minimum effects of mesh density on the observed stress drop.

Up to this point, elastic and plastic lattice rotations were allowed for the grains of the polycrystal (Eqs. (5)–(7)). Although with 3D-XRD average orientation or stress can be calculated for each grain, one may assume that highly localized stress or rotation fields may have caused such a stress drop. Our HR-EBSD on the same CPZr sample revealed large variation in stress and lattice rotation (Supplementary Fig. 2); since more than 950 IPs are assigned on average to a grain in the CPFE simulation, it is straightforward to examine the effects of such localized interactions. For the test model (M1), lattice rotations were turned off in our calculation—no change in stress drop was observed (Fig. 8b). This means that lattice rotations do not affect the stress drop at low applied strains. For the second case (M2), grain positions and shapes were kept the same, but random orientations were assigned to each grain in such way that the macroscopic texture of the whole aggregate represents a random texture (max and min of 0.7 and 1.4 multiples of random). The cumulative frequency of stress drop for this case was 22%, which is a minor change compared to the original 25%. This means that the effect of macroscopic texture is also relatively small. The results of these two cases imply that neighbouring grains must play a significant role. The most interesting results were captured for the case, where a self-consistent type approach was tested. For this case (M3), while the macroscopic texture was kept the same, grains were fragmented and the fragments were randomly dispersed into the bulk, i.e., random positions were assigned to the individual elements of each grain (Fig. 8c). For this case, individual elements of each grain interact with as many grains as possible, i.e., this method mimics the self-consistent type models, where each grain interact with a homogenous medium that represents the entire aggregate except the grain under study. Interestingly, no stress drop is observed for M3 model. This reflects the importance of incorporating the true effects of microstructure or grain–grain interactions into crystal plasticity models as neglecting those would result in extracting inaccurate single-crystal parameters and misinterpretation of stress relaxation that originate from other sources such as twinning. In order to understand the effects of element clustering on the results, every two elements of a grain were clustered and similar to the M3 model, they were randomly distributed into the bulk. This model is called M4. Results from this model do not show any significant change comparing to M3 confirming the significant effect of grain–grain interactions. The effects of such interactions on the formation of twins in CPTi were previously studied using 3D-XRD method^17^; however, results shown in this paper show a stress drop in the absence of twins.

**Fig. 9:**
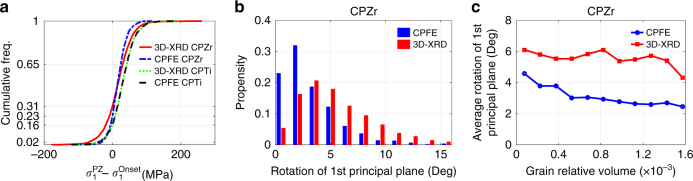
Investigation of the 1st principal stresses and their associated 1st principal planes. **a** Cumulative frequency of 1st principal stress drop for CPTi and CPZr. PZ refers to plastic zone, where maximum strains of 1.2% and 0.94% were applied to CPZr and CPTi samples, respectively. **b** Rotation of 1st principal planes of each grain from onset to maximum load in CPZr. **c** Average rotation of 1st principal planes as a function of grain-measured relative volume

So far the variation of *σ*_33_ is discussed, however, at the grain level, there is a 3D stress state. Due to the local constraints applied by the neighbouring grains, all of the six stress components can be non-zero, hence, in this section the variation of the first principal stress *σ*_1_ and the corresponding principal plane are investigated. It is shown in Fig. 9a, that 31% of the CPZr grains and 16% of CPTi grains measured by 3D-XRD undergo *σ*_1_ stress drop. Similarly, CPFE results show that 23% of CPZr grains and 16% of CPTi grains undergo *σ*_1_ stress drop. These results are very close to those reported for *σ*_33_ confirming the significant contribution of this stress component on the calculation of *σ*_1_. The effects of other stress components is more vivid when the rotation of the first principal plane is studied. It is shown in Fig. 9b that the first principal planes of most of the grains undergo less than 5° rotation from the onset of plasticity to the applied strain of 1.2%. It is interesting to report that there are some grains that experience more than 10° rotation in the very-small straining step. In Fig. 9c, these rotations are plotted against grain-measured relative volume, where it is shown that smaller grains undergo larger rotations. Such a large rotation of principal plane is purely induced by the neighbouring grains within the small grain, which has to accommodate sharp stress gradients from one grain boundary to another. This observation further confirms the significance of grain neighbour effects.

Deformation of CPZr and CPTi as two of the technologically important HCP polycrystals was studied through linking 3D-XRD experiments with CPFE modelling. In contrast to other available diffraction techniques, with 3D-XRD all of the components of the stress tensor can be extracted by taking into account the contribution of more than 90 crystal planes that appear as diffraction spots. This provides the opportunity of measuring 3D deformation states with confidence. Since grain relative volumes and COM positions can be measured by 3D-XRD, the grain morphologies were recovered through the use of weighted Voronoi tessellation. Each grain neighbours were subsequently determined and were categorised as hard neighbours (low *β*) and soft neighbours (high *β*).

Despite the comprehensive analysis of HCP polycrystals in the literature, it has never been reported that grain-resolved stresses along the loading direction can drop while applied stress increases. In this paper, we report such an observation in the absence of deformation twinning. It was shown that hard grains that are surrounded by hard neighbours experience a maximum stress drop. While high-resolution EBSD measurements confirm significant stress or rotation variation from one neighbour side to another, CPFE simulations confirmed the minimum effects of grain rotation on the stress drop. All of our modelling results suggest that elastic and plastic anisotropy of single crystals and strong grain–grain interaction are the main factors that contribute to the observed stress drop. This is significant as it suggests that study of deformation in HCP polycrystals demands the use of methods that provide proper grain–grain interactions.

## Methods

### 3D-XRD experiment

The experiment was conducted at the ID-11 of the European Synchrotron Radiation Facility (ESRF), Grenoble, France. The samples were mounted on an Admet tensile rig that could apply load uniaxially along the *z*-axis. The macroscopic applied load was measured by a load cell attached to the tensile rig, while the macroscopic strain was measured using two different methods: by tracking two silver wires glued to the sample and determining their precise position using the incident X-ray beam and by digital image correlation tracking changes in the position of the wires using optical images. Both CPTi and CPZr samples were deformed under strain control at an applied strain rate of 2.64 × 10^−5^ s^−1^. Diffraction measurements were conducted at four different steps (Fig. 2c): preload, onset of plasticity, maximum applied strain (*ε* = 1.2% for CPZr, and *ε* = 0.94% for CPTi), and finally unload. At each measurement step after aligning the sample, the centre of the probed volume was re-calculated by determining the current position of the silver wires and apply the necessary movements in the *z*-direction to follow the same volume. At each loading step, diffraction patterns were acquired using a monochromatic X-ray beam with the energy of 78.39 and 41 keV for CPZr and CPTi, respectively. In order to measure the state of the deformation in 3D, the sample was rotated about the *z*-axis so that various crystallographic planes of the crystals were brought into the diffraction condition (Fig. 1a). Due to constraints applied by the pillars of the tensile rig, it was not possible to scan the sample over the entire rotation range of 360°. For this experiment, sample and the loading stage were firstly rotated from −234.5 to −125.5°, and then from −54.5 to 54.5° both with the rotation step of 0.25°. The exposure time for collecting diffraction images with 2048 × 2048 pixels was 0.25 s for each rotation step.

In this article, *z*-axis represents loading direction, *x*-axis represents X-ray beam direction and *y*-axis is the cross-product of the other two axes. Stresses are labelled as 11, 22 and 33 where 1, 2 and 3 represent *x*-, *y*- and *z*-axis, respectively.

### Analysis of 3D-XRD diffraction patterns

The post processing of the measured data was mainly done by the use of ImageD11 and the subroutines embedded into Fable (https://sourceforge.net/p/fable/wiki/Home/). After calculating the pattern background and subtracting it from diffraction patterns, a series of peak searches were performed by applying a series of threshold intensity levels of 100, 200, 400, 800, 1600, 3200, 6400, 12,800 to determine the position of the measured peaks. Once diffraction peaks were identified, they were indexed and assigned to grains. This was done by cross correlating peaks from different rings using the hkl tolerance of 0.01. The indexed grains were used to refine parameters which in turn allowed further diffraction peaks to be indexed and additional grains determined. The global geometry parameters (i.e., parameters such as detector tilts, centre of the detector and sample to detector distance) were established by the use of diffraction patterns from a Ceria calibration sample. Grains with more peaks were then used to refine crystal parameters for the CPZr or CPTi test pieces. For final tuning of the parameters, the grain maps measured at the preload were used in the FitAllB program^30^. Once the final parameters were identified, they were used to re-index grains from preload and to find grains and their state of deformation at the onset of plasticity, and the two subsequent loading steps.

In order to match grains from each loading step, the rigid body movement of the whole-scanned volumes was first determined and corrected. This was done by comparing the COM of common grains from any two steps. Common grains are any grain pairs from two steps that not only have small misorientation and small movement in their COMs, but also more than six of their immediate neighbouring grains can be matched across the two loading steps. Once the common grains were identified, their COMs were used to fix rigid body movement of the whole-scanned volume. At this stage, it was possible to compare and match the grains of any two loading steps.

### Crystal plasticity finite element model

A series of models were generated using the weighted Voronoi tessellation. These models and the results of mesh convergence at the macroscopic and local level are shown in the Supplementary Figs. 3 and 4. For simulating the experiment, each model was deformed at the same strain rate that was used in the experiment. Periodic boundary conditions were applied on each of the surfaces of the simulation cube following the method described in Abdolvand et al.^25^ A tensile elongation was applied along the vertical *z*-axis at a rate matched to the experiment, while in the transverse *x*- and *y*-axes the contraction was left free and the model allowed to relax to zero transverse net force. Since both CPTi and CPZr samples were annealed and recrystallised before the in situ experiments, the effects of thermal residual stresses that can develop as a results of this process were taken into account. The thermal coefficient of expansion used in simulations for CPZr is (5.3, 5.3, 10.1) × 10^−6^ 1/°C along crystal (*a*, *b*, *c*) directions^7^ and for CPTi is (9.5, 9.5, 5.6) × 10^−6^ 1/°C^31,32^.

A crystal plasticity user material (UMAT) subroutine that was developed by Abdolvand et al.^25^ was used for simulating deformation of each grain. The UMAT solves constitutive equations and links to ABAQUS FE program, which solves force equilibrium and compatibility equations. At the beginning of each time increment, Abaqus passes strain and time increment data into the UMAT in which the new state of stress, solution-dependent state variables and the Jacobian matrix $\left( {\frac{{\partial {\mathrm{\Delta }}{\boldsymbol{\sigma }}}}{{\partial {\mathrm{\Delta }}{\boldsymbol{\varepsilon }}}}} \right)$ will be calculated and returned to the FE solver. The total strain increment (Δ**ε**) can be decomposed to the elastic part (Δ**ε**^**el**^) and the plastic (Δ**ε**^**pl**^) part. For the samples used in this study, the *c*-axis of the HCP polycrystals is mostly in compression, and since the deformation is applied to small strains, the effects of twinning are neglected; hence, the plastic strain rate can be calculated from the slip rate $\left( {\dot \gamma ^\alpha } \right)$:

2 $$\begin{array}{l}{\dot{\mathrm \varepsilon }}^{{\bf{pl}}} = \mathop {\sum }\limits_{\alpha = 1}^{N^{spl}} {\bf{P}}^{\bf{\alpha }}\dot \gamma ^{\bf{\alpha }} \\ {\bf{P}}^{\bf{\alpha }} = \mathrm{sym}\left( {{\bf{S}}^{\bf{\alpha }}} \right)\mathrm{where}\,{\bf{S}}^{\bf{\alpha }} = {\bf{d}}^{\bf{\alpha }} \otimes {\bf{n}}^{\bf{\alpha }}\end{array}.$$

In which **P**^**α**^ is the symmetric part of the Schmid tensor (**S**^**α**^) for the slip system *α*, **d**^**α**^ is the direction of the slip and **n**^**α**^ is the normal to the slip plane. Time integration of Eq. (2) will return plastic strain increment. The slip rate of the slip system *α* can be calculated using Eq. (3)^33^:

3 $$\dot \gamma ^\alpha = \dot \gamma _0\left| {\frac{{\tau ^\alpha }}{{g^\alpha }}} \right|^n\mathrm{sign}\left( {\frac{{\tau ^\alpha }}{{g^\alpha }}} \right),$$

where $\dot \gamma _0$ is a reference shear strain rate, *τ*^*α*^ is the resolved shear stress acting on the slip system *α* and *g*^*α*^ is the current CRSS of the slip system *α*. The shear stress acting on each slip system can be calculated from the Kirchoff stress (**Ψ**) through the following equation:

4 $$\tau ^\alpha = {\bf{P}}^{\bf{\alpha }}:{\bf{\Psi }}.$$

The Jaumann rate of Kirchoff stress $\left( \hat {\bf{\Psi }} \right)$ is related to the elastic part of the rate of deformation $\left( {{\dot{\bf D}}^{{\bf{el}}}} \right)$ and the elastic stiffness tensor $\left( {\Bbb C} \right)$ as:

5 $$\hat {\bf{\Psi }} = {\Bbb C}:{\dot{\mathbf D}}^{{\bf{el}}}\,{\mathrm{where}}\,\hat {\bf{\Psi }} = {\dot {\bf{\Psi }}} - {\dot{\bf \Omega }}^{{\bf{el}}}\,{\bf{\Psi }} + {\bf{\Psi }}{\dot{\bf \Omega }}^{{\bf{el}}},$$

where $\left( {{\dot{\bf \Omega }}^{{\bf{el}}}} \right)$ is the elastic part of the rotation tensor. The deformation and the rotation rates are correlated to the symmetric and the asymmetric parts of the velocity gradient (**L**) as:

6 $$\left( {{\dot{\bf D}}^{{\bf{el}}} + {\dot{\bf D}}^{{\bf{pl}}}} \right) + \left( {{\dot{\bf \Omega }}^{{\bf{el}}} + {\dot{\bf \Omega }}^{{\bf{pl}}}} \right) = \mathrm{sym}\left( {\bf{L}} \right) + \mathrm{asym}\left( {\bf{L}} \right).$$

and the plastic part of the rotation increment is correlated to the plastic shear rate and asymmetric part of the Schmid tensor (**W**^**α**^):

7 $${\dot{\bf \Omega }}^{{\bf{pl}}} = \mathop {\sum }\limits_{\alpha = 1}^{N^{spl}} {\bf{W}}^{\bf{\alpha }}\dot \gamma ^\alpha.$$

The strength of each slip system (Eq. (3)) is assumed to follow an extended Voce hardening:

8 $$g^\alpha = g_0^\alpha + \left( {g_1^\alpha + \theta _1^\alpha \Gamma } \right)\left( {1 - \mathrm{exp}\left( { - \frac{{\theta _0^\alpha \Gamma }}{{\it g_1^\alpha }}} \right)} \right),$$

where *g*^*α*^ is the CRSS of the slip system *α*, $g_0^\alpha$ is the initial CRSS, *Γ* is accumulated shear on all slip/twin systems, $\theta _0^\alpha$ is the initial hardening rate, and $g_1^\alpha$ and $\theta _1^\alpha$ determine asymptotic characteristics of hardening.

The elastic modulus of the single-crystal CPZr used in this study is the one determined by Fisher and Renken^34^: *C*_11_ = 143.5 GPa, *C*_33_ = 164.9 GPa, *C*_12_ = 72.5 GPa, *C*_13_ = 65.4 GPa and *C*_44_ = 32.1 GPa. Similarly, the elastic modulus of CPTi is *C*_11_ = 162.4 GPa, *C*_33_ = 180.7 GPa, *C*_12_=92 GPa, *C*_13_=69 GPa and *C*_44_=46.7 GPa.

### High-resolution EBSD

Data collection was carried out in a Zeiss MERLIN Field Emission Gun Scanning Electron Microscope (FEG-SEM) with 20 keV beam energy, 15 nA current and working distance of 18 mm. Kikuchi patterns were collected using a high-resolution EBSD detector. The EBSD detector was set at about 18 mm distance where 30 patterns, acquired from different sections of the sample, were used for final tuning of the sample to doctor distance. Each pattern was collected over 200 ms of exposure time while no gain was applied; detector binning of 2 × 2 was used to acquire 800 × 600 pixels Kikuchi patterns.

Elastic strain and lattice rotations were calculated by cross correlating the collected Kikuchi patterns. This was done by subdividing each pattern into 128 × 128 pixel images picked from the middle of the Kikuchi pattern, from the perimeter of the circle centred around the middle of the pattern, and some random positions within the pattern. About 20 sub-patterns were selected randomly, while 19 patterns were selected from the perimeter of the circle; with the central sub-pattern, all in all, 40 sub-patterns were used for the cross-correlation. For each grain, a point was selected as the reference point. It was assumed that the state of stress at the reference point is relatively low and uniform. All of the patterns collected within a grain were subsequently cross-correlated with the reference pattern from which relative elastic strains and lattice rotations were calculated^35^.

### Data availability

The data that support the findings of this study are available at Zenodo.org with the identifier 'doi: 10.5281/zenodo.1042139'. These data will also be made accessible on the Oxford Research Archive (www.ora.ox.ac.uk). All other supporting data from this study are available within the article and its Supplementary Materials file, or from the corresponding author upon reasonable request.

## Electronic supplementary material

Supplementary Information
