# Supplementary material for: Strong grain neighbour effects in polycrystals
Source: Nat Commun. 2018 Jan 12;9:171. doi: 10.1038/s41467-017-02213-9 (PMC5766582; doi:10.1038/s41467-017-02213-9)
Supplement: Supplementary file 1 — Supplementary Information [file 41467_2017_2213_MOESM1_ESM.pdf]

Supplementary Materials

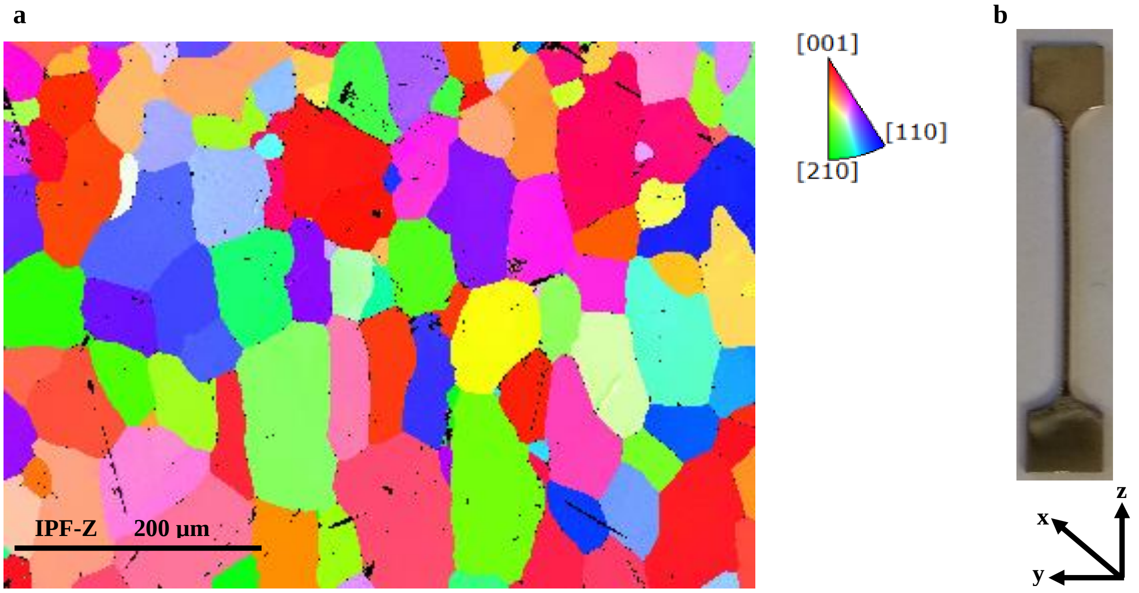

Supplementary Figure 1. Microstructure of the CPZr sample used for the 3D-XRD experiment. (a) An EBSD map of the undeformed sample with the color legend given in the right hand side of the figure. Colors are coded with respect to inverse pole figure z and the scale bar represent 200 μm. (b) The CPZr sample used in 3D-XRD experiment with the lab coordinate system shown in the bottom of the picture; z-axis is the loading direction and the sample gauge length is 20 mm.

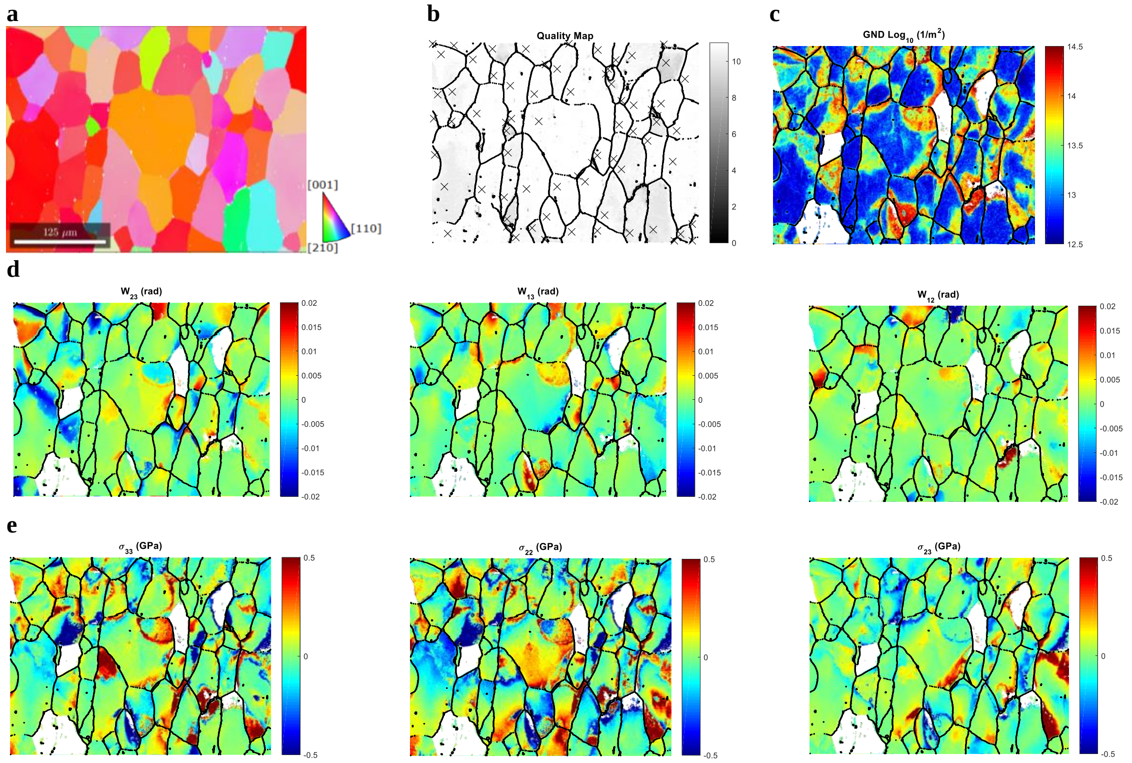

Supplementary Figure 2. (a) An EBSD map of the deformed CPZr sample. The scale bar represent 125 μm. (b) A map representing the quality of the measured Kikuchi patterns with red crosses representing reference points assigned to each grain for the relative rotation and stress calculation provided in (d) and (e). (c) The measured geometrically necessary dislocation density (GND). (d) The measured elastic lattice rotations. (e) The measured in-plane stresses.

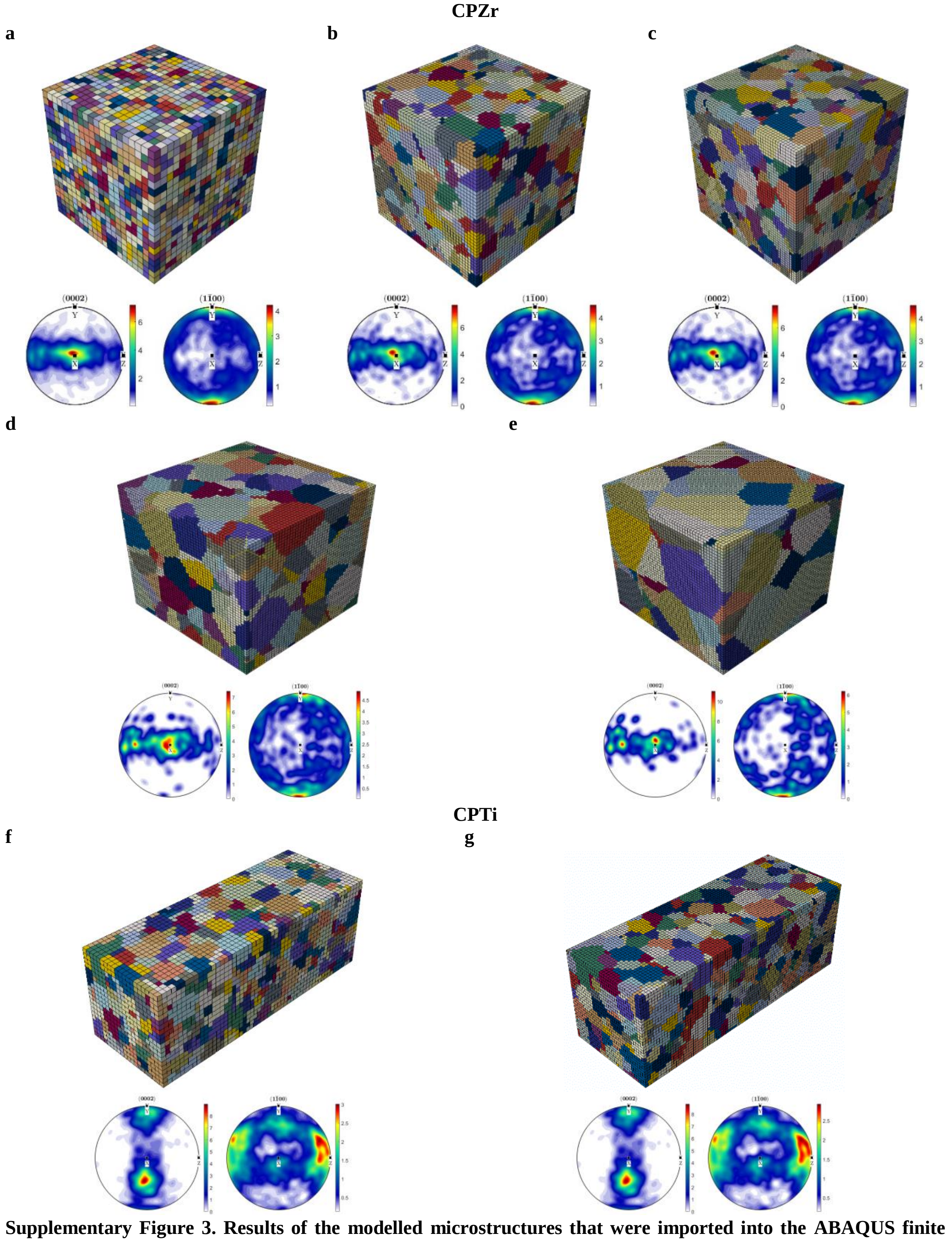

Supplementary Figure 3. Results of the modelled microstructures that were imported into the ABAQUS finite element solver. (a) Model-17 used for simulating 2963 grains; (b) Model-5 and (c) model-4 used for simulating 1038 grains of CPZr sample. The pole figure that each model represents is shown below the model and random colors were assigned to elements to distinguish different grains. In the model (a) a cube of 340^3 μm^3 was meshed while in (b) and (c) a cube of 200^3 μm^3 was meshed. These cubes were meshed with the step size of 17, 5, and 4 μm, respectively. Number of integration points assigned to each grain (IP/grain) on average are 22, 493, and 963, respectively. In (d) a cube of 150^3 μm^3 was meshed at the step size of 3 μm and in (d) a cube of 100^3 μm^3 was meshed at the step size of 2 μm. Number of simulated grains in (d) and (e) are 469 (2132 IP/grain) and 177 (5650 IP/grain), respectively.

(d) CPFE models of CPTi sample with (d) coarse mesh and (e) fine mesh. Pole figure that each model represents is shown below the model. A cube of 800^2 x 2200 μm^3 were meshed at the step size of (f) 40 μm and (g) 25 μm.

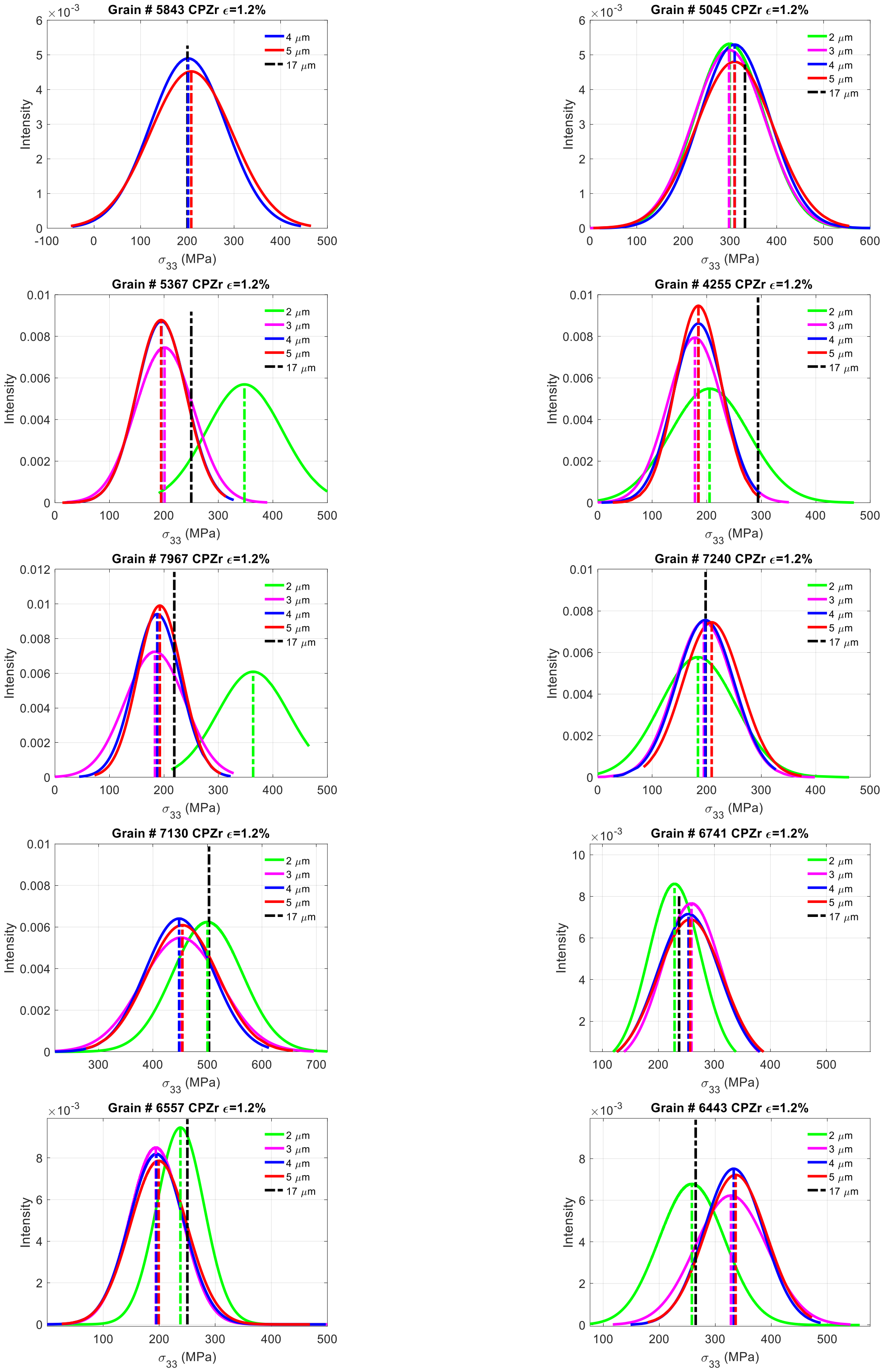

Supplementary Figure 4. Mesh convergence test at the grain level for the CPZr models showed in the Supplementary Fig. 3. Grain IDs are shown at the top of each figure and results are for σ_{33} stress component. 2, 3, 4, 5, and 17 refer to the step sizes used for meshing the cube, i.e. the smaller the number the more elements assigned to each grain. The deviation observed for the model 2 μm is due to the low number of grains used in this model (just 177 grains), i.e. grains do not see as many neighbors as they do in other models.

The solid lines represent variation of stress within the grains and the middle dashed lines represent the average stress calculated for the grains of each model.

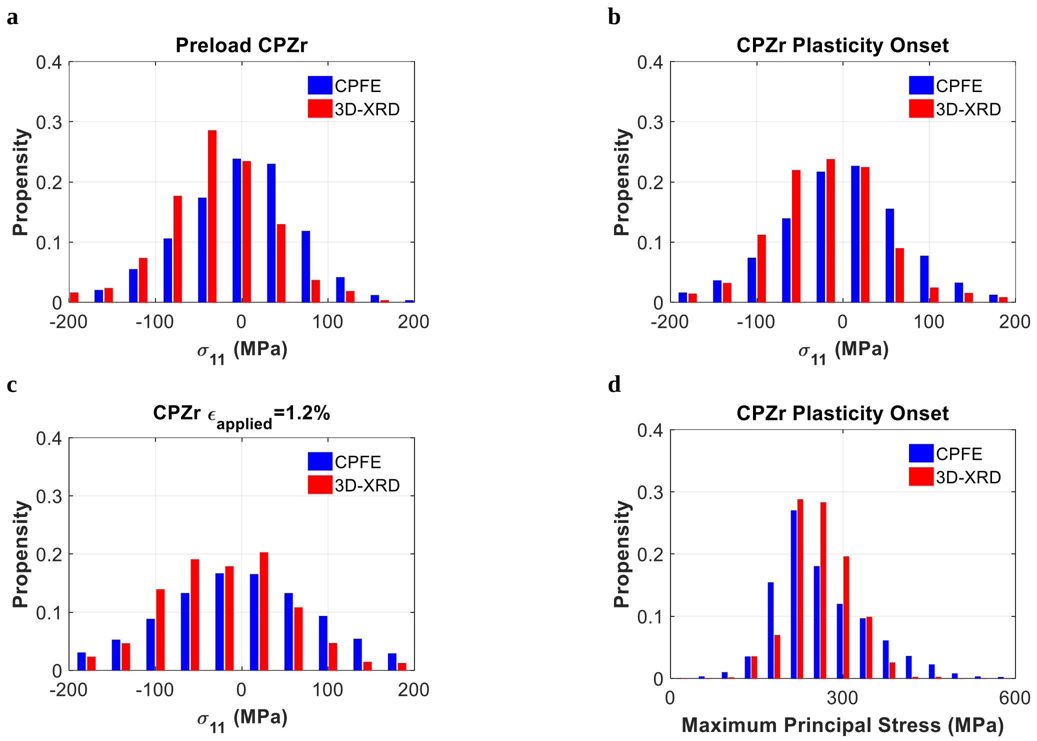

Supplementary Figure 5. Comparison between 3D-XRD measured and CPFE-NSH simulated values for σ_{11} (a) at the preload, (b) onset of plasticity, and (c) applied strain of 1.2%. (d) Measured and calculated maximum principal stresses at the onset of plasticity.

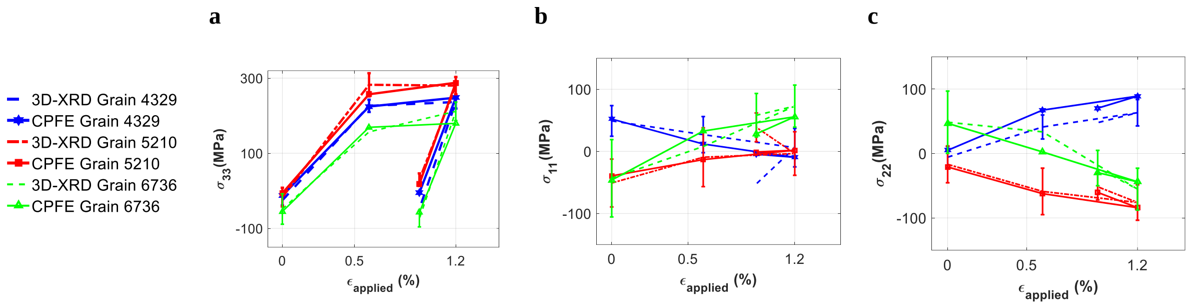

Supplementary Figure 6. Comparison between 3D-XRD measured and CPFE-NSH simulated values for avergae stress within selected grains of CPZr: (a) σ_{33}, (b) σ_{11}, (c) σ_{22}.

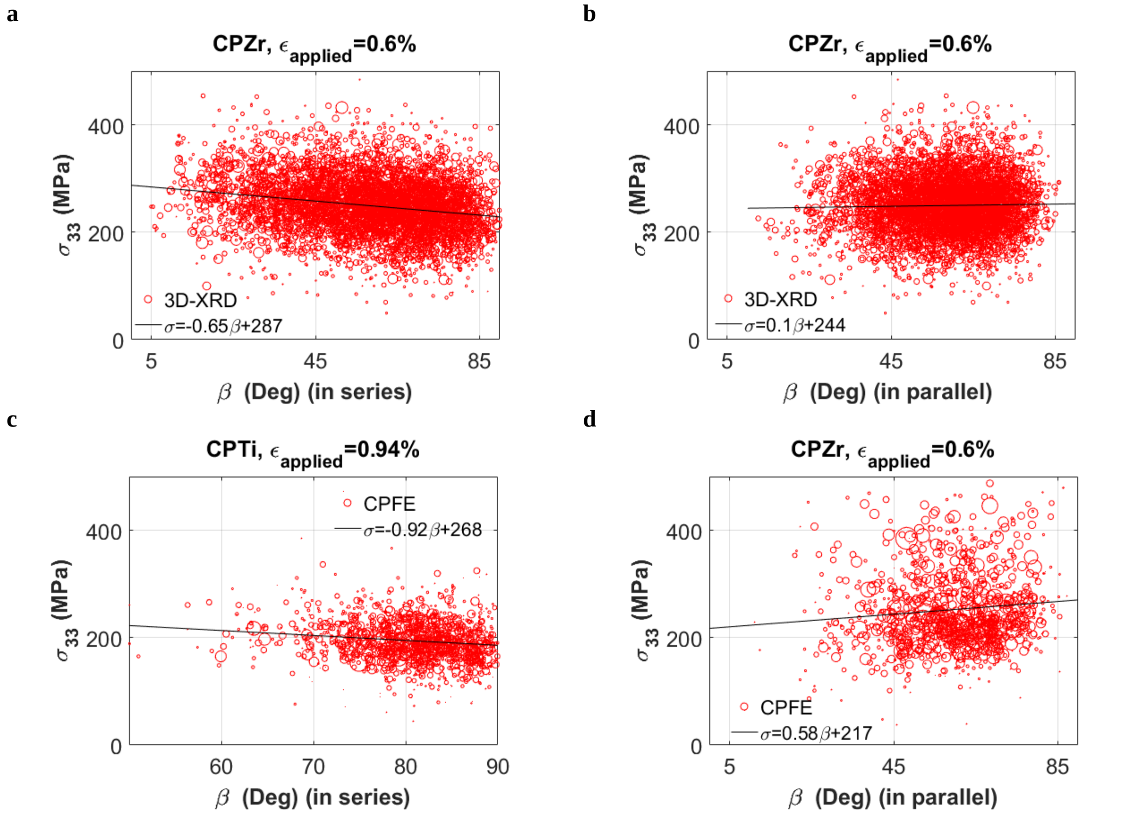

Supplementary Figure 7. 3D-XRD measured σ_{33} for CPZr at the onset of plasticity for grains (a) in series and (b) in parallel. Examples of CPFE results for (c) CPTi grains acting in series and (d) CPZr grains acting in parallel.

Supplementary Table 1. Chemical composition of the CPZr and CPTi samples

Zr	C	Hf	Fe	Cr	N	O	H
Balance	250 ppm	2500 ppm	200 ppm	200 ppm	100 ppm	1000 ppm	10 ppm
Ti	Fe	O_2	N_2	C			
Balance	o.35%wt	700 ppm	35 ppm	0.01 wt%			

Supplementary Table 2. single crystal properties of CPZr used for the model SH[25]

	$\boldsymbol{n}$*	$\dot{\boldsymbol{\gamma}}_0(\boldsymbol{s}^{-1})$**	$\boldsymbol{g}_0^{\alpha}$ (GPa)	$\boldsymbol{g}_1^{\alpha}$ (GPa)	$\boldsymbol{\theta}_0^{\alpha}$ (GPa)	$\boldsymbol{\theta}_1^{\alpha}$ (GPa)
**Prism**	20	3.5×10^{-4}	0.12	0.33	0.01	0
**Basal**	20	3.5×10^{-4}	0.168	0.22	0.05	0
**Pyramidal**	20	1.0×10^{-4}	0.331	0.27	0.62	0.28

*, ** in all of the simulations the same n and $\dot{\gamma}_0$ were used
